# Editorial: Glycobiology and glycosylation: deciphering the secrets of glycans in humans and pathogens

**DOI:** 10.3389/fimmu.2025.1560135

**Published:** 2025-02-03

**Authors:** Parham Jabbarzadeh Kaboli, Charalampos Proestos

**Affiliations:** ^1^ Department of Biochemistry, Faculty of Medicine, Medical University of Warsaw, Warsaw, Poland; ^2^ Laboratory of Food Chemistry, Department of Chemistry, School of Sciences, National and Kapodistrian University of Athens, Athens, Greece

**Keywords:** glycosylation, immune response, cancer immunotherapy, glycan structures, disease mechanisms

## Introduction

Glycosylation, the process of adding carbohydrates to proteins, is a fundamental biological process with far-reaching implications for human health and disease. These glycan modifications play critical roles in numerous cellular processes, including protein folding, cell signaling, and immune recognition. Their dysregulation is implicated in various diseases, including cancer, infectious diseases, and autoimmune disorders (1, 2).

One striking example of glycosylation’s importance is in the cancer immunotherapy field. The effectiveness of cancer treatments, especially immunotherapies like anti-PD-L1 monoclonal antibodies (e.g., atezolizumab), can be significantly impacted by altered glycosylation patterns on tumor cells (3, 4). These alterations can shield tumor cells from immune surveillance and dampen the response to immunotherapy. For instance, atezolizumab’s withdrawal from breast cancer treatment due to limited efficacy highlights the challenges posed by altered glycosylation (5). Within this landscape, the galectin family of proteins, particularly galectin-9, emerges as a critical player in cancer progression and resistance to therapy, underscoring the intricate link between glycosylation and immune evasion, where galectin-9 acting as a potential barrier to effective immunotherapy, including treatments like atezolizumab (6, 7).

Recognizing the increasing significance of glycobiology in health and disease, *Frontiers in Immunology* has published a Research Topic titled *“Glycobiology and Glycosylation: Unraveling the Mysteries of Glycans in Humans and Pathogens.”* This Research Topic of insightful articles delves into the intricate world of glycans, with each article offering a unique perspective on the connection between glycobiology and therapeutic strategies:

## Changes in glycan signatures in chondrocytes

Homan et al. analyze how *in vitro* passaging of chondrocytes alters their glycan profiles and subsequently influences their interaction with the immune system. Their results indicate that passaged chondrocytes provoke a more intense pro-inflammatory response in macrophages, marked by increased IL-6 and nitric oxide production, compared to non-passaged chondrocytes. This research highlights the dynamic nature of glycan expression and its potential implications for cell-based therapies, such as cartilage transplants.

## IgA glycosylation patterns in COVID-19

Potaczek et al. investigate the glycosylation patterns of IgA antibodies in the plasma of critically ill COVID-19 patients. They found notable differences in IgA glycosylation, characterized by reduced sialylation and heightened galactosylation in patients experiencing acute respiratory distress syndrome (ARDS). These changes were linked to the increased formation of neutrophil extracellular traps (NETs), suggesting a possible connection between IgA glycosylation and thromboembolic complications in severe COVID-19 cases.

## Heparan sulfate biosynthesis in prostate cancer

Grigorieva et al. explore the complex regulation of heparan sulfate biosynthesis in fibroblasts co-cultured with normal or cancerous prostate cells. Their findings suggest that cancer cells do not influence this regulatory mechanism, while normal epithelial cells downregulate genes associated with heparan sulfate synthesis in fibroblasts. This disparity in cellular communication may contribute to the unchecked growth and progression of prostate cancer, indicating the therapeutic potential of targeting glycosaminoglycan pathways.

## Galectins in cell adhesion to vascular endothelium

Souchak et al. provide an in-depth review of the roles of galectins, specifically Gal-3, -8, and -9, in facilitating circulating cell adhesion to the vascular endothelium. They examine the mechanisms by which galectins promote adhesion through direct interactions and indirect signaling that enhances the expression of adhesion molecules on endothelial cells. This adhesion process is crucial for various physiological and pathological events, including immune cell trafficking, stem cell homing, and the metastasis of circulating tumor cells (CTCs). The review also considers potential therapeutic strategies to address inflammation and cancer spread by disrupting galectin-mediated adhesion.

## Glycan-based scaffolds for targeted drug delivery in cancer therapy

Qin et al. investigate the innovative application of glycan-based scaffolds and nanoparticles as targeted drug delivery systems in cancer treatment. They emphasize the unique properties of glycans—such as specificity, versatility, and low immunogenicity—that make them ideal for this purpose. Glycan-based delivery systems have the potential to enhance drug efficacy while minimizing off-target effects by explicitly targeting tumor cells or tissues. The authors also address the challenges in designing and producing these complex scaffolds, calling for further research and development in this promising field.

## Recognition of hypervirulent *Klebsiella pneumoniae*

Campanero-Rhodes et al. examine how the innate immune system detects hypermucoviscous variants of *Klebsiella pneumoniae*. Their research focuses on the interactions between bacterial surface glycans and immune lectins, particularly from the Siglec and galectin families. The findings highlight the critical role of glycan structures in pathogen recognition and the intricate relationship between host immunity and bacterial virulence. Understanding these interactions is vital for developing novel strategies to combat infections caused by hypervirulent strains.

## O-GlcNAc transferase in pulmonary fibrosis

Vang et al. present pioneering research on the function of O-GlcNAc transferase (OGT) in regulating collagen deposition and the resolution of fibrosis in idiopathic pulmonary fibrosis (IPF). Their findings reveal significantly elevated levels of OGT in IPF patients, implicating OGT in the excessive collagen accumulation characteristic of this severe lung disease. Through single-cell RNA sequencing and experimental models, they demonstrate that inhibiting OGT can effectively reverse collagen expression and accumulation. This discovery positions OGT as a potential therapeutic target for IPF and other fibrotic diseases characterized by excessive extracellular matrix deposition.

## Common glycoepitopes in cancer cells and viruses

Roy explores the shared abnormal glycosylation patterns in cancer cells and enveloped viruses. These atypical glycosylation profiles, often resulting from deficiencies in glycosyltransferase activities, mutations, or overexpression of chaperones, contribute to unique glycoepitopes in cancer cells. These tumor-associated carbohydrate antigens have emerged as promising targets for developing glycoconjugate vaccines. Notably, the N-linked glycans on viral glycoproteins often resemble those found on normal host cells, while specific O-linked glycans closely mirror those present on tumor cells. This intriguing overlap suggests glycoconjugate vaccines could stimulate robust immune responses against cancer and viral infections.

## Conclusion

The diverse articles featured in this Research Topic significantly enhance our understanding of glycobiology and its complex implications for human health and disease mechanisms. These studies illuminate how subtle changes in glycan structures can profoundly affect immune responses, cancer progression, and interactions between hosts and pathogens. We are confident that all contributions within this Research Topic will serve as a vital resource for researchers in the field and inspire further exploration into the captivating world of glycans.
